# Regulating Effect of Cytochrome *b_5_* Overexpression on Human Breast Cancer Cells

**DOI:** 10.3390/molecules27144556

**Published:** 2022-07-17

**Authors:** Xin-Yi Tong, Xin-Zhi Yang, Shu-Qin Gao, Xiao-Juan Wang, Ge-Bo Wen, Ying-Wu Lin

**Affiliations:** 1 School of Chemistry and Chemical Engineering, University of South China, Hengyang 421001, China; 20202005110239@stu.usc.edu.cn (X.-Y.T.); 2004000789@usc.edu.cn (X.-J.W.); 2 Hengyang Medical College, University of South China, Hengyang 421001, China; 201511310019@stu.usc.edu.cn (X.-Z.Y.); 2014001853@usc.edu.cn (S.-Q.G.); wengebo@usc.edu.cn (G.-B.W.); 3 Key Lab of Protein Structure and Function of Universities in Hunan Province, University of South China, Hengyang 421001, China

**Keywords:** Cytochrome *b_5_*, Cytochrome *c*, protein-protein interactions, breast cancer cells, co-immunoprecipitation

## Abstract

Imbalance in the cellular redox system is thought to be associated with the induction and progression of breast cancers, and heme proteins may regulate the redox balance. Cytochrome *b_5_* (Cyt *b_5_*) is a small mitochondrial heme protein. Its function and regulating mechanism in breast cancer remain unknown. In this study, we elucidated the level of endogenous oxidative stress in breast cancer cells, MCF-7 cells (hormone receptor-positive cells) and MDA-MB-231 cells (triple-negative cells), and investigated the difference in Cyt *b_5_* content. Based on the low content of Cyt *b_5_* in MDA-MB-231 cells, the overexpression of Cyt *b_5_* was found to regulate the oxidative stress and apoptosis cascades, including ERK1/2 and Akt signaling pathways. The overexpressed Cyt *b_5_* MDA-MB-231 cells were shown to exhibit decreased oxidative stress, less phosphorylation of ERK1/2 and Akt, and less cleavage of caspases 3 and 9 upon treatment with H_2_O_2_, as compared to those of normal MDA-MB-231 cells. Moreover, the overexpressed Cyt *b_5_* most likely functioned by interacting with its protein partner, Cyt *c*, as suggested by co-immunoprecipitation studies. These results indicated that Cyt *b_5_* has different effects on breast cancer cells of different phenotypes, which provides useful information for understanding the multiple roles of Cyt *b_5_* and provides clues for clinical treatment.

## 1. Introduction

Breast cancer is known to be one of the “hidden killers” of women, which ranks first among female malignant tumors. According to the latest statistics in 2018, there were more than 2.08 million new cases all around the world, with 15% of breast cancer deaths in women [1,2]. Mitochondrial dysfunction and oxidative stress are the key factors inducing diseases such as aging and cancers [3]. It was estimated that ~90% reactive oxygen species (ROS) are generated from the mitochondria, including superoxide anions (O_2_^•^^−^), hydrogen peroxides (H_2_O_2_) and hydroxide radicals (^•^OH), etc. [4]. The levels of ROS are critical for a variety of biological processes, including mitochondrial and plasma membrane function, cell signaling, and immune response [5]. Meanwhile, the imbalance of the redox system affects the ROS content and leads to changes in cell signaling, oxidative damage, and cytotoxicity [5,6,7,8,9]. Recent studies also indicate that persistent over-produced ROS may induce cellular adaptation, which usually occurs in cancers and plays an important role in the development of cancers. Furthermore, excessive ROS may induce cell deaths because of DNA damage and lipid peroxidation [6,7,8,9].

Imbalance in the redox system is also thought to be associated with the induction and progression of breast cancers. Cancer cells usually exhibit a higher level of ROS compared to normal cells, due to mitochondrial dysfunction, and increased inflammatory activity [10], while cancer cells may also increase antioxidant defense which will balance oxidative status [11]. The role of ROS and oxidative stress both in the initiation and development of breast cancer is supported by much evidence [11]. In addition, H_2_O_2_ induces apoptosis by regulating the mitogen-activated protein kinase (MAPK) signaling [12].

Human cytochrome *b_5_* (Cyt *b_5_*) is a small heme protein located at the outer mitochondrial membrane [13], which is involved in some oxidative reactions in biological tissues, as an electron transfer component, and participates in the regulation of cellular redox balance [14]. Moreover, Cyt *b_5_* may exist as dimers or tetramers in living organisms [15]. Although Cyt *b_5_* itself has no direct enzymatic catalysis, it may interact with other heme proteins, such as Cyt *c*, Cyt P450 and myoglobin, playing certain physiological functions [16,17]. In addition, Cyt *b_5_* is considered to be involved in apoptosis because it may interact with Cyt *c* to inhibit apoptosis [14]. Nevertheless, the function of Cyt *b_5_* and its regulating mechanism in breast cancer remain unknown.

In this study, we aimed to evaluate the regulating effect of Cyt *b_5_* on the oxidative stress level in human breast cancer cell lines, such as MDA-MB-231 and MCF-7 cells, which are representative of the triple-negative breast cancer cell and hormone receptor-positive breast cancer cell, respectively [18]. We also investigated the relationship between oxidative stress in these two types of cells and the overexpression of Cyt *b_5_*, aimed at clarifying the characteristics of triple-negative breast cancer from the perspective of oxidative stress. The overexpressed Cyt *b_5_* was shown to regulate oxidative stress and apoptosis cascade, including ERK1/2 and Akt signaling pathways. This study will provide useful information for understanding the regulatory role of Cyt *b_5_* in maintaining the balance of the redox system in cancer cells.

## 2. Results and Discussion

### 2.1. Endogenous Oxidative Stress Level and Expression of Cyt b_5_ in MDA-MB-231 and MCF-7 Cells

**Figure 1:**
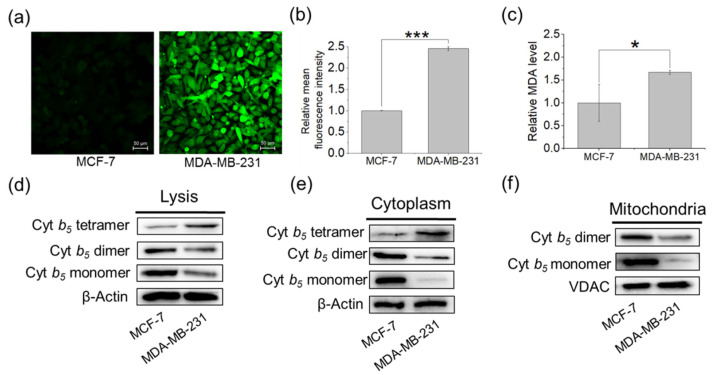
Analysis of oxidative stress and Cyt *b_5_* content in MDA-MB-231 and MCF-7 cells. (**a**) Analysis of ROS levels in MDA-MB-231 and MCF-7 cells by treating with DCFH-DA reagent. (**b**) ROS detection was quantified by using ImageJ software and the optical densitometry was reported. (*) *p* < 0.05; (***) *p* < 0.001. (**c**) Analysis of MDA levels in MDA-MB-231 and MCF-7 cells by using lipid peroxidation MDA assay kit. (*) *p* < 0.05; (***) *p* < 0.001. (**d**–**f**) Representative western blot of Cyt *b_5_* in MDA-MB-231 and MCF-7 cells in the whole cell lysis, cytoplasm, and mitochondria. β-Actin was used as an internal control in lysis and cytoplasm and VDAC was used as an internal control in mitochondria.

To evaluate the endogenous oxidative stress levels of breast cancer cells, we selected MDA-MB-231 and MCF-7 cell lines as the representative models of triple-negative and estrogen receptor-positive (ER+) breast cancer cells, respectively. First, we analyzed ROS levels in MDA-MB-231 and MCF-7 cells by using a fluorogenic probe, 2,7-Dichlorodihydrofluorescein diacetate (DCFH-DA), which is commonly used to detect total intracellular ROS content. As shown in Figure 1a,b, the fluorescence microscopy highlighted that the green fluorescence intensity of DCFH-DA was significantly higher in MDA-MB-231 cells compared to that in MCF-7 cells, which indicated that MDA-MB-231 cells are characterized by higher levels of total ROS content than MCF-7 cells. As further shown in Figure 1c, the malondialdehyde content in MDA-MB-231 cells was found to be higher than that in MCF-7 cells, suggesting a higher degree of lipid oxidation in the triple-negative MDA-MB-231 cells. These observations agreed with the difference as reported previously [19]. Triple-negative breast cancer cells depend on a high level of ROS for survival and, thus, an antioxidant therapy was developed to induce cell death in triple-negative cells instead of ER+ cell lines [10]. 

In principle, different oxidative stress levels may lead to diverse expressions of antioxidant proteins. Thus, we analyzed the expression of Cyt *b_5_*, belonging to the antioxidant system, in both MDA-MB-231 and MCF-7 cells. Figure 1d–f presented the representative western blot results, which showed that the antioxidant protein Cyt *b_5_* was expressed both in MCF-7 and MDA-MB-231 cells. Most Cyt *b_5_* molecules existed as monomers and dimers in MCF-7 cells, whereas the tetramer of Cyt *b_5_* in MDA-MB-231 cells was more than that in MCF-7 cells. Moreover, different multimeric forms of Cyt *b_5_* were found in various species and tissues and the oligomerization of Cyt *b_5_* in vivo might be influenced by many factors. For example, the aggregated forms of sheep Cyt *b_5_* were affected by ionic strength and pH value [20]. It should be noted that Cyt *b_5_* tetramer might not perform normal physiological functions [20], and the monomeric form of Cyt *b_5_* is believed to be the active form [21,22,23,24,25]. These studies coincided with our experimental results, which suggested that more Cyt *b_5_* tetramer and less monomer in MDA-MB-231 cells might result in a higher degree of oxidative stress.

### 2.2. Effects of Overexpressed Cyt b_5_ on the Endogenous Oxidative Stress Levels

**Figure 2:**
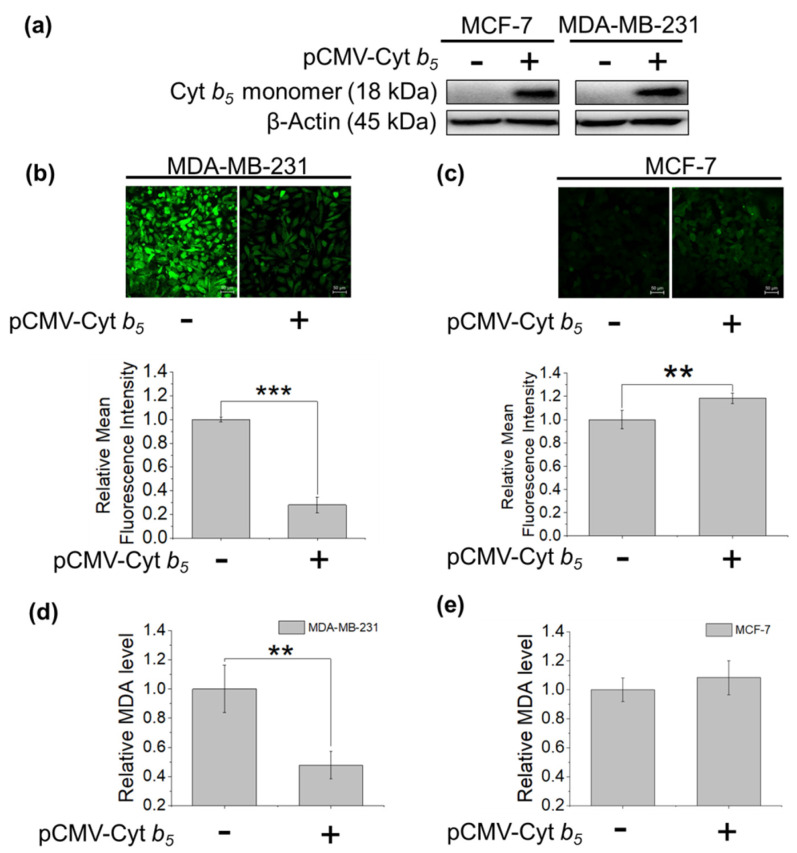
Overexpression of Cyt *b_5_* and its regulating effect on the oxidative stress in MDA-MB-231 and MCF-7 cells. (**a**) Validation of the overexpression of Cyt *b_5_* in MDA-MB-231 and MCF-7 cells by western blot. (**b**,**c**) Detected ROS levels of MDA-MB-231 (**b**) and MCF-7 (**c**) cells by treatment with DCFH-DA reagent. (**) *p* < 0.01; (***) *p* < 0.001. (**d**,**e**) Analysis of MDA levels in MDA-MD-231 (**d**) and MCF-7 cells by using lipid peroxidation MDA assay kit. (**) *p* < 0.01; (***) *p* < 0.001.

To evaluate whether overexpression of Cyt *b_5_* affects the levels of endogenous oxidative stress in MDA-MB-231 and MCF-7 cells, we constructed plasmids containing the Cyt *b_5_* gene with a flag-tag (pCMV-Cyt *b_5_*) and performed transient transfections of the two breast cancer cells to overexpress Cyt *b_5_*. As shown in Figure 2a for the western blot test, the flag-tag was prominently expressed in both cells, demonstrating the successful overexpression of Cyt *b_5_*. The flag-tagged Cyt *b_5_* was observed only at around 20 kDa, which indicated that the overexpressed Cyt *b_5_* exhibited in a monomer form. Detection with DCFH-DA reagent further showed that MDA-MB-231 cells with overexpressed Cyt *b_5_* had a lower ROS level than the ordinary MDA-MB-231 cells (Figure 2b). In studying the extent of lipid oxidation, the marker product of malondialdehyde was detected (Figure 2d). The results showed that the amount of malondialdehyde in normally cultured MDA-MB-231 cells was higher than that in MDA-MB-231 cells with overexpressed Cyt *b_5_*, which indicated that the degree of lipid peroxidation of MDA-MB-231 cells was decreased after the overexpression of Cyt *b_5_*. These results further suggested that the overexpression of Cyt *b_5_* reduced the degree of oxidative stress in MDA-MB-231 cells. However, different results were observed for those of MCF-7 and Cyt *b_5_*-overexpressed MCF-7 cells, with a slight increase in the ROS level (Figure 2c) and lipid oxidation (Figure 2e). Based on the fact that the Cyt *b_5_* content in MCF-7 cells was relatively high, the overexpression of Cyt *b_5_* might not have much effect on its oxidative stress, although the mechanism remains unknown. Meanwhile, the Cyt *b_5_* content in MDA-MB-231 cells was relatively low and the overexpression of Cyt *b_5_* might have some effects on its oxidative stress. Therefore, our subsequent research focused on the overexpression of Cyt *b_5_* in MDA-MB-231 cells and its effects. 

It should be noted that although Cyt *b_5_* does not have a direct catalytic function in cells, it is involved in many redox equilibrium processes as an electron transporter, such as the interaction with Cyt *c* and P450, which are needed to maintain metabolic homeostasis in mammalian cells [26]. It has been proposed that the presence of Cyt *b_5_* can decrease the release of O_2_^•−^ and H_2_O_2_ [27]. Chen et al. showed that the overexpression of Cyt *b_5_* in drosophila retina completely inhibits blue light-induced lipid peroxidation and retinal degeneration [28], which suggests that Cyt *b_5_* is a neuroprotective factor against light-induced oxidative damage, particularly lipid peroxidation. Cyt *b_5_* may function by supporting antioxidant recycling, providing a strategy to prevent oxidative stress in aging photoreceptors. On the other hand, alteration of the normal function of Cyt *b_5_* may lead to an increase in ROS. As shown by Kocadal et al. [29], inorganic mercury disrupts the oxidative phosphorylation and electron transport pathways at the ubiquinone-Cyt *b_5_* site, which induces ROS production and accumulation. 

### 2.3. Effects of Overexpressed Cyt b_5_ on the Redox Balance Ability of MDA-MB-231 Cells

**Figure 3:**
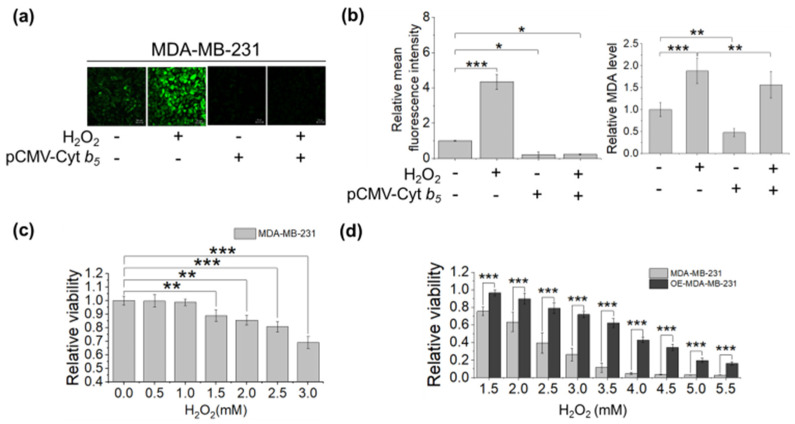
Overexpression of Cyt *b_5_* in MDA-MB-231 cells and treatment with H_2_O_2_. (**a**) Detected ROS levels in MDA-MB-231 cells by treating with DCFH-DA reagent. (**b**) Analysis of MDA levels in MDA-MD-231 cells by using lipid peroxidation MDA assay kit. (**c**) Analysis of MTT assays in H_2_O_2_-treated MDA-MB-231 cells with 0–3.0 mM H_2_O_2_. (**d**) Analysis of MTT assays in H_2_O_2_-treated MDA-MB-231 cells and H_2_O_2_ (1.5–5.5 mM)-treated MDA-MB-231 cells with overexpressed Cyt *b_5_*. (*) *p* < 0.05; (**) *p* < 0.01; (***) *p* < 0.001.

To explore the processing capacity of MDA-MB-231 cells with overexpressed Cyt *b_5_* for exogenous H_2_O_2_, we performed four treatments on the cells with or without H_2_O_2_. The ROS content of each group was detected by using the DCFH-DA probe. As shown in Figure 3a, the fluorescence intensity in the H_2_O_2_ group increased significantly compared to that of the control group, which indicated that H_2_O_2_ was indeed able to cause an increase in ROS content in MDA-MB-231 cells. A slight decrease in fluorescence was observed for the overexpressed group relative to the control group (Figure 3a), indicating that the overexpression of Cyt *b_5_* was able to reduce ROS content. After the stimulation of Cyt *b_5_*-overexpressed MDA-MB-231 cells by 1 mM H_2_O_2_ for 12 h, the fluorescence intensity was similar to that of MDA-MB-231 cells with overexpressed Cyt *b_5_* and was much lower than H_2_O_2_ group, which demonstrated that the overexpressed Cyt *b_5_* moderately alleviated the increased ROS content, as induced by exogenous H_2_O_2_. 

As further detected of malondialdehyde levels in each group, it showed a significant increase in the malondialdehyde content for the H_2_O_2_ group relative to the control group (Figure 3b), which suggested that H_2_O_2_ is indeed capable of causing an increase in lipid oxidation in MDA-MB-231 cells. Moreover, the malonaldehyde content was decreased in the overexpression group relative to the control group, suggesting that the overexpression of Cyt *b_5_* could reduce the degree of lipid peroxidation. After the stimulation of Cyt *b_5_*-overexpressed MDA-MB-231 cells by 1 mM H_2_O_2_ for 12 h, the malondialdehyde content was higher than that of the ordinary MDA-MB-231 cells and was lower than that of H_2_O_2_-stimulated cells, respectively, which indicated that the overexpressed Cyt *b_5_* moderately, although not completely, alleviated the increased degree of lipid oxidation as induced by exogenous H_2_O_2_. 

The sensitivity of cells to H_2_O_2_ was further determined by treating both cell lines with increasing concentrations of H_2_O_2_ (0.5–3 mM). After the treatment with H_2_O_2_ for 12 h, an MTT assay was performed to monitor cell viability. As shown in Figure 3c, the relative viability of MDA-MB-231 cells was significantly decreased when treated with 1.5 mM H_2_O_2_. The sensitivity of MDA-MB-231 and Cyt *b_5_*-overexpressed MDA-MB-231 cells was then determined by treating both cell lines with increasing concentration of H_2_O_2_ from 1.5 mM to 5.5 mM for 12 h. The results showed that the Cyt *b_5_*-overexpressed MDA-MB-231 cells were more resistant to high concentrations of H_2_O_2_ than the normal MDA-MB-231 cells (Figure 3d). In particular, at the concentration of < 4 mM, MDA-MB-231 cell viability was almost decreased to the minimum whereas the Cyt *b_5_*-overexpressed MDA-MB-231 cells were still at a high cell survival rate. These observations suggested that when MDA-MB-231 cells overexpress Cyt *b_5_*, the processing capacity for high concentration H_2_O_2_ stimulation is enhanced.

### 2.4. Effects of Overexpressed Cyt b_5_ on Akt/MAPK Signaling Pathway in MDA-MB-231 Cells

**Figure 4:**
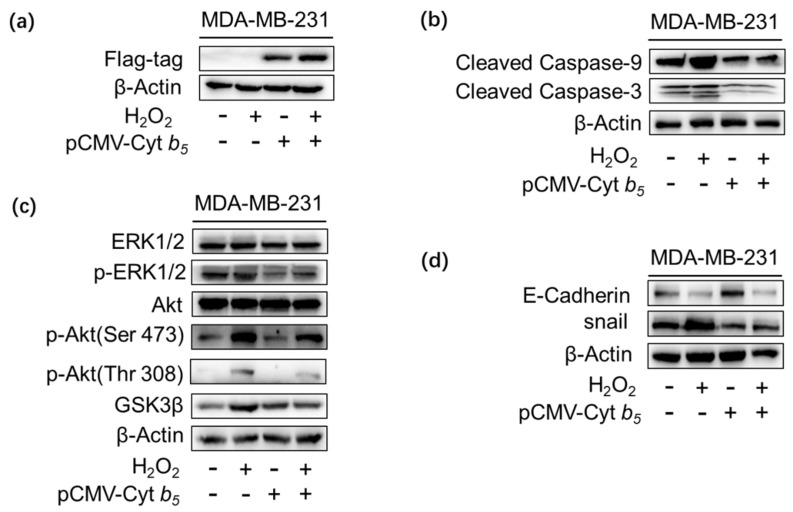
Effects of overexpression of Cyt *b_5_* on cell signaling pathways. (**a**) Significant expression of Flag-tag in MDA-MB-231 cells, as verified by western blot analysis. (**b**–**d**) Changes in apoptosis-related proteins (**b**), ERK1/2 and Akt signaling pathways (**c**), and snail/E-Cadherin signaling pathway (**d**), in MDA-MB-231 cells by western blot analysis.

Based on the sensitivity of MDA-MB-231 cells to H_2_O_2_, we chose 1 mM H_2_O_2_ to stimulate the cells to reach a state of oxidative stress without leading to cell death, so that we could observe the changes in the pre-apoptotic state. The cells were also divided into four groups and western blot test was conducted to probe the protein content differences. The results showed that the β-Actin levels were consistent across the four groups (Figure 4a–d), whereas significant expression of flag-tag was detected in two groups of Cyt *b_5_* overexpression (Figure 4a). Testing of apoptosis-related proteins was then performed. It revealed a clear cleavage of caspase-3 and caspase-9 in H_2_O_2_-treated MDA-MB-231 cells, which was increased by ~20% compared to the untreated groups, instead of in the overexpressed Cyt *b_5_* cells and H_2_O_2_-treated overexpressed Cyt *b_5_* cells (Figure 4b). Moreover, there was no significant difference in the total ERK1/2 and total Akt content for the four groups, whereas the p-ERK1/2 and p-Akt levels varied between different groups (Figure 4c). The p-ERK1/2 content of the H_2_O_2_-treated group increased by ~20% compared to the control group, and the p-ERK1/2 content of the overexpressed Cyt *b_5_* groups (treated/untreated with H_2_O_2_) was lower (~90%) than that of the H_2_O_2_-treated group. Similar results were also presented in the Akt signaling pathway (Figure 4c). These observations were consistent with reports from the literature. ROS can affect molecular pathways related to apoptosis, cell proliferation, and invasion. 

Subsequently, we detected tumor cell invasion and metastasis-related proteins in these four groups. As presented in Figure 4d, the stimulation of H_2_O_2_ resulted in a reduction by ~60% in E-cadherin compared to that of the control group. The Cyt *b_5_*-overexpressed MDA-MB-231 cells were characterized by ~15% more E-Cadherin and ~50% less snail compared to the normal MDA-MB-231 cells. The situation in the overexpressed Cyt *b_5_* group was similar to that of the Cyt *b_5_*-overexpressed MDA-MB-231 cells. The loss of E-cadherin and the epithelial-mesenchymal transition (EMT) are key steps in triple-negative breast cancer progression [30]. Similar H_2_O_2_-induced reduction in expression of E-Cadherin and increase in the expression of snail in breast cancer cells have been reported [31]. Snail is a downstream effector of ERK pathway, which inhibits the expression of E-cadherin and, thus, inhibits cell migration [32]. The present results showed that the overexpression of Cyt *b_5_* in MDA-MB-231 cells promoted the expression of E-Cadherin through the ERK/Snail/E-Cadherin signaling pathway, thereby reducing the invasion and metastasizing ability of breast cancer cells.

### 2.5. Possible Interactions between Overexpressed Cyt b_5_ and Cyt c in MDA-MB-231 Cells

**Figure 5:**
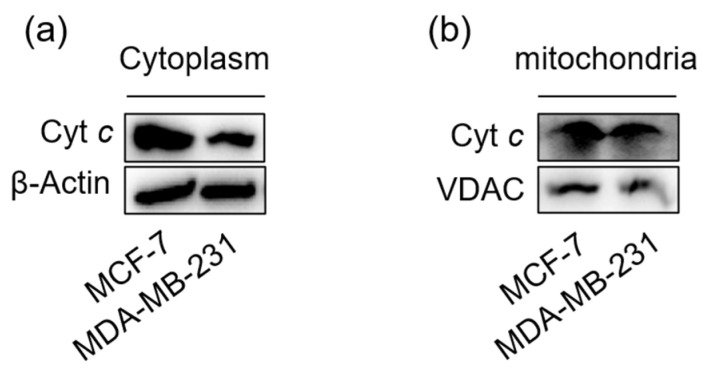
Expression levels of Cyt *c* in cytoplasm (**a**) and mitochondria (**b**) in MCF-7 cells and MDA-MB-231 cells, respectively, with those of β-Actin and VDAC shown for comparison.

It has been suggested that Cyt *b_5_* may interact with its protein partner, cytochrome *c* (Cyt *c*), and play an important role in vivo [14,33], as well as in vitro [34]. There are at least three binding modes of Cyt *b_5_*-Cyt *c* interactions, including the Salemme model (E44-K27, E48-K13, D60-K72, and propionate-K79) [35], the Northrup model (E48-K13, E56K-K87, D60-K86, and propionate-K72) [36], and the Huang model (E37-K86, E38-K72, and propionate-K13) [37]. As analyzed with western blot (Figure 5), both MCF-7 cells and MDA-MB-231 cells expressed Cyt *c*, whereas the expression level in MCF-7 cells was higher than that in MDA-MB-231 cells, as observed for both cytoplasm and mitochondria. The overexpressed Cyt *c* may regulate the ROS levels in the breast cancer cells, which may be related to protein reactivity, such as peroxidase activity and protein-protein interactions [38,39,40,41]. The mechanism still remained elusive.

**Figure 6:**
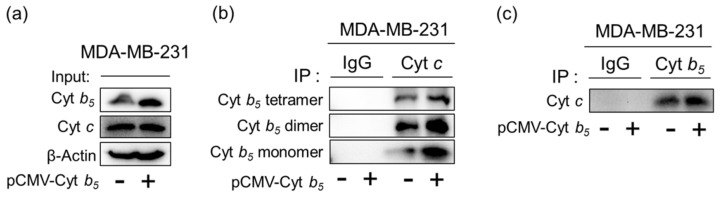
Co-IP verified the interaction of Cyt *b_5_*-Cyt *c* in MDA-MB-231 cells. (**a**) Cyt *b_5_* and Cyt *c* were co-expressed in MDA-MB-231 cells and in MDA-MB-231 cells with overexpressed Cyt *b_5_*. (**b**,**c**) Cell extracts were incubated with Cyt *c* antibody (**b**) and Cyt *b_5_* antibody (**c**). The immune complexes were precipitated by protein A/G beads and Cyt *b_5_*/Cyt *c* was analyzed with western blot.

To reveal whether the overexpressed Cyt *b_5_* interacts with Cyt *c* in MDA-MB-231 cells, we tested the combination of Cyt *b_5_* and Cyt *c* by co-immunoprecipitation (Co-IP) studies. As shown in Figure 6, the amount of the Cyt *b_5_*-Cyt *c* complex in the overexpressed Cyt *b_5_* group was significantly more than that of the control group, which suggested that the overexpressed Cyt *b_5_* in MDA-MB-231 cells binds Cyt *c* in large quantities, leading to relieving of oxidative stress by combination with Cyt *c*. After incubation with Cyt *c* antibody overnight, the Co-IP experiment also showed bands of Cyt *b_5_* tetramer and dimer in complex with Cyt *c* (Figure 6b), indicating interactions between the oligomers of Cyt *b_5_* and Cyt *c*. This observation suggested that the oligomerization of Cyt *b_5_* may not alter the structure of the heme-binding domain that interacts with Cyt *c* [42].

**Scheme 1:**
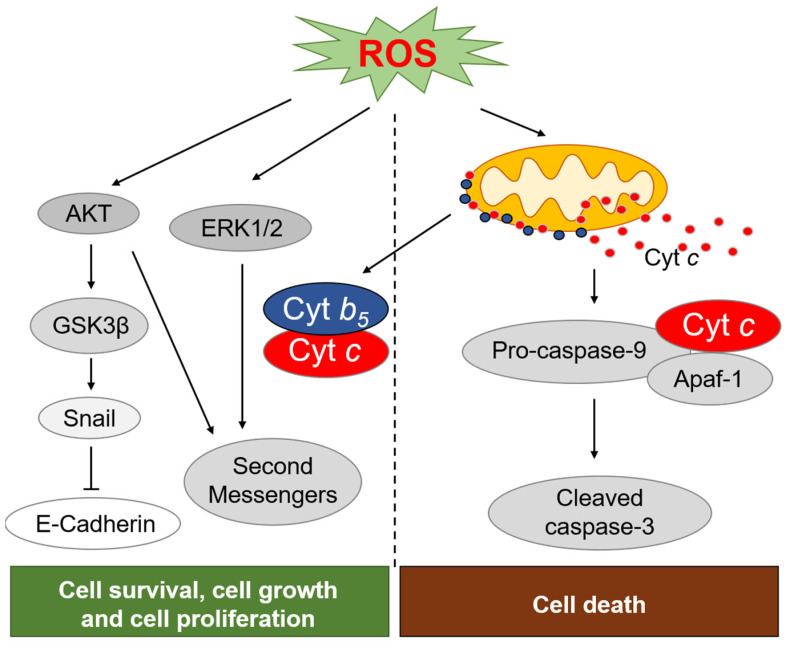
ROS and cell signaling in cell survival, cell growth and cell proliferation, as well as in cell death, as referenced to the literature [44].

Moreover, the interaction of Cyt *b_5_* with Cyt *c* may block the interaction of Cyt *c* with Apaf-1. Similar to Cyt *b_5_*, the Apaf-1 surface is enriched with negatively charged residues of Asp and Glu. As predicted by the simulated Apaf-1-Cyt *c* complex, Cyt *c* may use similar positively charged Lys residues to interact with Apaf-1 such as K27, K72, K79, K86 and K87 [43]. Therefore, we speculated that in MDA-MB-231 cells, when Cyt *b_5_* is overexpressed, Cyt *c* will interact with it during the process of release from mitochondria to the cytoplasm, which prevents the continued binding of Cyt *c* to APAF-1, thereby preventing the apoptosis cascade. As shown in Scheme 1, we summarized the oxidative stress in triple-negative breast cancer and proposed the possible effects of Cyt *b_5_*.

## 3. Materials and Methods

### 3.1. Materials

Malondialdehyde detection by lipid peroxidation MDA assay kit and ROS detection by reactive oxygen species assay Kit were provided by Beyotime Institute of Biotechnology (Nantong, Jiangsu, China). Polyvinylidene fluoride (PVDF) was from Millipore (Billerica, MA, USA). Dulbecco’s modified eagle medium (DMEM), fetal bovine serum (FBS), and phosphate-buffered saline (PBS) were products from Gibco (Australia). Lipofectamine™ 3000 was from Invitrogen (Thermo Fisher Scientific, Waltham, Massachusetts, USA). The primary antibodies for cleaved caspase-9, cleaved caspase-3, phospho-p44/42 MAPK (p-ERK1/2), AKT, Phospho-Akt (Thr308), Phospho-Akt (Ser473), DYKDDDDK Tag, snail and β-actin were purchased from Cell Signaling Technology (Danvers, MA, America). The primary antibodies for Cyt *c* and Cyt *b_5_* were purchased from Abcom (Cambridge Biomedical Campus, Cambridge, UK). 3-(4,5-dimethylthiazol-2-yl)-2,5-diphenyltetrazolium bromide (MTT) was from Aladdin (Shanghai, China). The phosphatase inhibitor cocktail and protein inhibitor cocktail were from Beijing ComWin Biotech Co., Ltd. (Beijing, China). Confocal laser imaging was recorded on the Zeiss LSM 880, Western blot analysis was performed on the Tanon 5200, and OD value measurement was detected using SpectraMax Absorbance Reader.

### 3.2. Cell Culture

We selected two human breast cancer lines MCF-7 and MDA-MB-231 as in vitro experimental models. Human breast cancer cell lines MCF-7 (Michigan Cancer Foundation-7, SCSP-531) and MDA-MB-231 (SCSP-5043) were obtained from the National Collection of Authenticated Cell Culture (Shanghai, China). Both cells were cultured in DMEM containing 4.5 g/L glucose, 10% (*v*/*v*) FBS in a humidified atmosphere with 5% CO_2_ at 37 °C. The medium was changed twice weekly and the cultures were split 1:3 each week for MCF-7 and twice a week for MDA-MB-231 cells, respectively.

### 3.3. Generation of an MDA-MB-231 Cell Line Overexpressing Cyt b_5_

Eukaryotic expression vector of pCMV-3Flag-Cyt *b_5_* was constructed by Genscript Biotech Corporation (Nanjing, Jiangsu, China). The full length of the human Cyt *b_5_* gene was cloned into the eukaryotic expression vector of pCMV-3Tag-1A and the plasmid containing Cyt *b_5_* gene was stored routinely in our laboratory. We seeded 1 × 10^5^ cells/well into a 6-well plate for transfection with pCMV-3Flag-Cyt *b_5_* plasmid using Lipofectamine™ 3000. The cells were cultured in DMEM containing 10% FBS and 1000 µg/mL G418 (MACKLIN reagent, Shanghai, China) for selection, and a stable Cyt *b_5_* expression cell line was obtained after four weeks.

### 3.4. Cell Viability Assay

Cell viability was measured by the MTT assay. The cultured cells were incubated with different concentrations (0.5–5.5 mM) H_2_O_2_ for 12 h, and then incubated with MTT reagent at 37 °C for 4 h in a humidified, 5% CO_2_ atmosphere. After the conversion of the water-soluble yellow dye MTT to an insoluble purple formazan by the action of SDH (succino-dehydrogenase) in mitochondrial, DMSO (dimethyl sulfoxide, Basel, Switzerland, Damas-Beta, China) was added to dissolve the formazan, and the amount was quantified by measuring the absorbance at 490 nm with a SpectraMax absorbance reader (Molecular Devices, San Jose, CA, USA). A control group was carried out without the addition of Cyt *b_5_* in the DMEM medium.

### 3.5. Lipid Peroxidation MDA Assay

Lipid peroxidation levels were measured by malondialdehyde (MDA) assay kit according to the technical instruction. According to the instructions, the cell sample and MDA detection working solution were prepared. The standard curve was produced by taking an appropriate amount of standard product after dilution. 0.1 mL of RIPA lysis buffer with 0.2 mL of MDA assay working solution was used as a blank control, and 0.1 mL of the sample with 0.2 mL of MDA assay working solution was used for the assay, by heating at 100 °C for 15 min. The samples were cooled to room temperature and centrifuged at 1000× *g* for 10 min at room temperature. An amount of 200 μL of the supernatant was added to the 96-well plate and subsequently determined by the absorbance at 532 nm using a microplate reader.

### 3.6. Intracellular ROS Detection

A ROS Assay kit (Beyotime Institute of Biotechnology, Nantong, Jiangsu, China) was used to determine the level of intracellular ROS. MDA-MB-231, MCF-7 cells and Cyt *b_5_* expression cell lines were seeded in Confocal Dish (Coverglass Bottom Dish) at a density of 8 × 10^4^ cells per well and treated with 1 mM H_2_O_2_ for 12 h at 37 °C [45]. The following steps were performed according to the manufacturer’s protocol. Briefly, adherent cells were washed with PBS, and each well was incubated with 1 mL 10 mM dichloro-dihydro-fluorescein diacetate (DCFH-DA) at 37 °C for 30 min. The wells were washed three times with PBS. The ROS in situ was observed using a fluorescence microscope (Zeiss LSM880). Fluorescence semi-quantitative analysis was performed using ImageJ software.

### 3.7. Preparation of Mitochondrial and Cytosolic Fractions

Mitochondrial and cytosolic fractions were isolated by using the Cell Mitochondria Isolation Kit (Beyotime Institute of Biotechnology, Nantong, Jiangsu, China). Briefly, the cells were washed with cold PBS, harvested by trypsin-EDTA solution, and collected by centrifuge. 1 mL mitochondrial isolation regent (with 1 mM PMSF) was added to resuspend the cells. The suspension was incubated on an ice bath for 20 min, which was then transferred to a proper-size EP tube for ultrasonic disruption of cells. The broken cells were centrifuged at 600× *g*, 4 °C for 10 min. Then the supernatant was transferred to another centrifugal tube by centrifuge for 10 min at 11,000× *g*, 4 °C. The precipitation was the isolated mitochondria and the supernatant was the cytoplasmic protein, respectively. All the reagents were provided by the cell mitochondria isolation kit.

### 3.8. Co-immunoprecipitation and Western Blot

Cells were collected and lysed. For immunoprecipitation, equal amounts of cells were incubated with primary antibodies at 4 °C for 12 h on a rotating incubator, and then the samples were further incubated with the 20 μL Protein A/G agarose (Beyotime Institute of Biotechnology, Nantong, Jiangsu, China) at 4 °C for 2 h rotation. Immunocomplexes were washed four times with PBST buffer before diluting with SDS-PAGE sample loading buffer. The proteins were separated by SDS-PAGE and transferred to PVDF membranes. The blots were incubated with primary antibody at 4 °C overnight, including anti-cleaved caspase-9 (CST), anti-cleaved caspase-3 (CST), anti-phospho-p44/42 MAPK (anti-p-ERK1/2) (CST), anti-AKT (CST), anti-phospho-Akt (Thr308) (CST), anti-phospho-Akt (Ser473) (CST), anti-DYKDDDDK Tag (CST), anti-snail (CST) and anti-β-Actin (CST), anti-Cyt *c* (Abcom) and anti-Cyt *b_5_* (Abcom). Then, the blots were incubated in secondary antibody after proper washing. The Immunoreactive proteins were developed and detected in Tanon 5200 Series Fully Automatic Chemiluminescence/Fluorescence Image Analysis System using enhanced chemiluminescence (ECL). Experiments were replicated at least three times, and representative blots were shown. The quantification of proteins was analyzed using ImageJ software.

### 3.9. Statistical Analysis

Analysis was performed in triplicate and representative images reported. Western blot and ROS detection were quantified by using ImageJ software and optical densitometry was reported. Data are mean ± standard deviation (SD) from three independent experiments, which were analyzed statistically by one-way analysis of variance (ANOVA) with the SPSS software. *p* values less than 0.05 were considered statistically significant.

## 4. Conclusions

In summary, this study revealed significant differences in the oxidative stress levels in two different types of breast cancer cells, MCF-7 cells (hormone receptor-positive cells) and MDA-MB-231 cells (triple-negative cells). The overexpression of Cyt *b_5_* in MDA-MB-231 cells was found to reduce the degree of oxidative stress, alter the apoptosis cascade, most likely by interacting with Cyt *c*, and increase the resistance to H_2_O_2_ via the ERK1/2 and Akt signaling pathways. This study indicates that Cyt *b_5_* plays an important role in maintaining the balance of the redox system in cancer cells, with different effects on breast cancer cells of different phenotypes. These observations provide useful information for understanding the multiple roles of Cyt *b_5_* and provide clues for clinical treatment.

## Data Availability

The datasets for this manuscript can be obtained from the corresponding author upon reasonable request.

## Sample Availability

Samples of Cyt *b*_5_, MCF-7 and MDA-MB-231 cells are available from the authors.
